# Enhancing students’ learning in problem based learning: validation of a self-assessment scale for active learning and critical thinking

**DOI:** 10.1186/s12909-015-0422-2

**Published:** 2015-08-26

**Authors:** Umatul Khoiriyah, Chris Roberts, Christine Jorm, C. P. M. Van der Vleuten

**Affiliations:** 1 Medical Education Unit (MEU) Fakultas Kedokteran UII, Jl.Kaliurang Km 14.5 Ngaglik, Sleman, Yogyakarta, 55584 Indonesia; 2 Sydney Medical School - Northern, the University of Sydney, Hornsby Kur-ring-gai Hospital, Palmerston Road, Hornsby, NSW 2077 Australia; 3 Sydney Medical School, Edward Ford Building A27, the University of Sydney, Sydney, NSW 2006 Australia; 4 Maastricht University, Educational Development and Research, P.O. Box 616, Maastricht, 6200 MD The Netherlands

## Abstract

**Background:**

Problem based learning (PBL) is a powerful learning activity but fidelity to intended models may slip and student engagement wane, negatively impacting learning processes, and outcomes. One potential solution to solve this degradation is by encouraging self-assessment in the PBL tutorial. Self-assessment is a central component of the self-regulation of student learning behaviours. There are few measures to investigate self-assessment relevant to PBL processes. We developed a Self-assessment Scale on Active Learning and Critical Thinking (SSACT) to address this gap. We wished to demonstrated evidence of its validity in the context of PBL by exploring its internal structure.

**Methods:**

We used a mixed methods approach to scale development. We developed scale items from a qualitative investigation, literature review, and consideration of previous existing tools used for study of the PBL process. Expert review panels evaluated its content; a process of validation subsequently reduced the pool of items. We used structural equation modelling to undertake a confirmatory factor analysis (CFA) of the SSACT and coefficient alpha.

**Results:**

The 14 item SSACT consisted of two domains “active learning” and “critical thinking.” The factorial validity of SSACT was evidenced by all items loading significantly on their expected factors, a good model fit for the data, and good stability across two independent samples. Each subscale had good internal reliability (>0.8) and strongly correlated with each other.

**Conclusions:**

The SSACT has sufficient evidence of its validity to support its use in the PBL process to encourage students to self-assess. The implementation of the SSACT may assist students to improve the quality of their learning in achieving PBL goals such as critical thinking and self-directed learning.

## Background

Problem Based Learning (PBL) is a learner-centred method, which has been implemented in many medical programs worldwide for over four decades. PBL has positive impacts on student learning and stimulates students to become lifelong learners [1]. However concerns have been raised about ‘signs of erosion’ in the original PBL process [2], which have had negative impacts on both learning processes and outcomes [2–4]. Students, teachers, and curriculum designers can all contribute to degradation in the quality of PBL. Students in particular, may perform hapzardly in the tutorial process and deviate from the intended procedures, which were developed by Faculty based on the underlying philosophy of PBL. For example, whilst brainstorming is essential for activating students’ prior knowledge, it tends to be shortened or sometimes even skipped during PBLs [2].

Two broad types of dysfunctional student behaviour during the tutorial process have been identified [4]. Individual dysfunctional behaviour refers to students’ performances that do not support the collaborative learning process. Students may be too quiet or dominant, lack commitment, experience personality clashes, or arrive late. Group dysfunctional behaviour is related to disorganise tutorial activities, especially groups taking shortcuts in the tutorial process.

As the PBL tutorial does not always work optimally as a learning method that fosters active, constructive, and goal-directed learning, comprehensive corrective actions that are in line with PBL philosophy are needed. Dolmans et *al*., [3] offer three solutions: using regular evaluation to improve group performance, stimulating elaboration, and using more formative rather than summative assessment. Moust et *al.* [2] also recommend improving the learning environment by giving more support to students to become self-directed learners, and by introducing self-assessment to induce student learning.

Self-assessment supports learners in exploring their own strengths and weaknesses in learning [5, 6]. Self-assessment is necessarily a comparative process, with the student comparing their own performance to specific standards or to previous performances or to the performance of others. Self-assessment is a key component of “assessment as learning,” [7] where students apply self-regulatory processes in their learning such as setting goals, selecting learning strategies, assessing learning progress, evaluating information from feedback, and then making improvements in their learning processes for the next time.

Self-assessment seems ideally suited for implementation into the PBL tutorial [8]. Some literature exists on self-assessment in the PBL tutorial process; however, most employ a self-assessment tool that has not been evaluated for this construct [9–12]. Moreover most research focuses on the ‘accuracy’ of self-assessment and shows that students are inaccurate in assessing their own performance in the PBL tutorial [11, 13, 14]. Whilst this may be true, there has been little research on the ways in which self-assessment might enhance student learning [15]. For this to happen, the implementation of a self-assessment tool requires scaffolding, for example by enhancing students’ awareness of the value of self-assessment, providing continuous feedback to students, and improving the design of the self-assessment tool around a specific task with specific objectives [16, 17]. Self assessment tools should also be designed with the broader context of learning in mind, and not focus on a specific domain such as knowledge acquisition [18]. The PBL self-assessment tools reported in previous research [10, 11, 13] have not been constructed or implemented using such approaches.

We aimed to fill this gap by developing a valid self-assessment tool to be used in the PBL tutorial setting, which we named the Self-assessment scale for active learning and critical thinking (SSACT). The tool was specifically designed to enhance student learning by promoting the self-assessment of students’ performance during the PBL process phases of problem analysis, self-directed learning, and reporting [19]. The purpose of this research was to investigate the validity of the SSACT in the context of the PBL tutorial by determining its internal structure [20–22]. We investigated the internal structure through assessing the factorial validity, scale stability and internal consistency of the SSACT [23].

## Methods

**Fig. 1:**
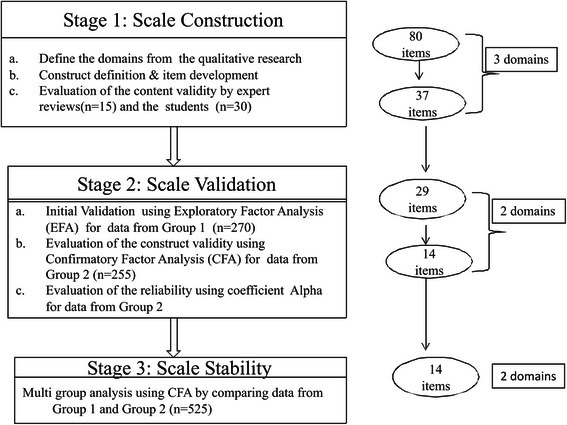
The flowchart of the development and validation stage of SSACT

The development of the SSACT consisted of three stages namely; 1) scale construction 2) scale validation and 3) investigation of scale stability (see Fig. 1). The study was conducted in the academic year 2013–2014 at the Faculty of Medicine, Islamic University of Indonesia (FM IUI), which employs PBL in the pre-clinical phase (year 1–4) of a 6 year course. Study participants were students and tutors who had experienced PBL.

Ethical approval was obtained from the University of Sydney, where the first author was a doctoral student (approval numbers 2013/1057, 2014/191 and 2014/344). On receipt of this approval, the Dean of the FM IUI endorsed this study. Written informed consent was obtained from the students and tutors at the FM IUI, who participated in this study.

*Scale construction* was based on the results of qualitative research undertaken with 10 students and 10 tutors with at least one-year experience of PBL tutorials, at the FM IUI. Semi-structured in-depth interviews [24] were conducted in Bahasa Indonesia by the first author (UK) using questions which explored their experiences in conducting the PBL tutorial, and their perspectives of self-assessment. The data from the interviews was transcribed, coded and analysed using thematic analysis to identify the contributing domains pertinent to self-assessment [25]. From this analysis, three themes were identified. These were initially labelled as: self-directed learning, teamwork, and reasoning skills, and were used as the preliminary domains in the subsequent process of scale construction stage.

The first author subsequently created an initial pool of 80 items by combining interpretation of student and staff perspectives of the PBL process, understandings of the literature, and incorporation of items from existing tools [26]. Each of the initial pool items reflected specific characteristics one of the three preliminary domains underlying the tool. For instance, the item “I applied various learning strategies during independent study“ reflected the students’ capability to apply appropriate self directed learning strategies. All of these 80 items were created by the first author in two languages, Bahasa Indonesia and English, and were reviewed by CR and CJ for clarity and English language. The Indonesian version of the scale was applied in this research. However, the development process of the scale was conducted both in Bahasa Indonesia and English using a de-centering approach. In this approach both of these two languages were equally important and the modification process was conducted simultaneously [27].

To assure its content validity, we used a panel of experts (n = 15) from Indonesia and Australia to review the initial set of 80 items [28]. This panel consisted of a psychologist who had expertise in measurement, ten medical educators who had experience as PBL tutors, and four non-medical educators who were familiar with PBL and had been a tutor for at least one year. The reviewing process was conducted through an online questionnaire, which was completed in two stages [29]. In the first stage, the experts were asked to match each item with the three domains provided, in order to determine the agreement among the experts. In the second stage, they evaluated the clarity of each item to ensure the wording was unambiguous. Items in which the representativeness score was less than 70 % were removed from the scale or were rewritten based on the feedback of the experts in the second stage.

Thirty students were then invited to complete the revised self-assessment tool and to give feedback regarding ambiguity in the items, the clarity of the instructions to complete the scale, and the time to complete the self-assessment tool. Consequently, the questionnaire was further refined and reduced to 37 Likert scale items, across the three preliminary domains. A 7-point Likert scale (*1 = ‘not very true of me’ to 7 = ‘very true of me’*) was chosen since a response scale with up to 7 points offers better reliability, validity and discriminant power than a scale with less points [30]. This questionnaire was designed to be completed by the students at the end of the tutorial meeting by reflecting on their tutorial performance in the previous unit or scenario.

*Scale validation* involved inviting students from the third and fourth years (n = 270), labelled Group 1, to use the 37 item scale resulting from the scale construction stage. Completed data was available from 256 questionnaires (94.8 %). These were analysed with exploratory factor analysis (EFA) using the oblique rotation method. Item redundancy was determined based on the following assumptions: a) the loading factor for each item > 0.5, b) an average corrected item-to- total correlation > 0.35, c) the average of the inter-item correlation > 0.20, d) no overlap among the items or wording redundancy, and e) relevancy to the theory underlying the tool [31]. Consequently it appeared that two factors under laid the scale. These were named ‘active learning’ and ‘critical thinking,’ and the scale was further reduced to 29 items.

The self-assessment tool of 29 items was then distributed to students from the first and second year (n = 255), labelled Group 2, to further validate the scale by confirming the factor structure. Of 255 students, 250 students returned the self-assessment tool; however because of incomplete data 238 questionnaires (93,1 %), were used in the analysis. The scale validation was conducted through Confirmatory Factor Analysis (CFA) using AMOS™ software to assess the dimensionality as a feature of the internal structure of the measurement scale [26, 32, 33]. Scale dimensionality refers to the homogeneity of the items and the factors underlying a construct. The dimensionality of the tool was evaluated using selected criteria of fit indices to assess whether the model was a close fit to the data or not. The criteria employed were: a) The goodness of fit index (GFI) > 0.9; b) Adjusted goodness of fit index (AGFI) > 0.8 [34]; c) the root mean square error approximation (RMSEA) < 0.1 [35]; d) the p value should be significant and the chi square divided by degrees of freedom < 3 [36]; e) the Tucker Lewis coefficient (TLI); and f) comparative fit index (CFI) > 0.90 [37]. After model fit was established, the internal consistency of the scale was measured using Cronbach’s alpha for the total scale and for each of the two sub-scales [38].

*Scale stability* was established by cross-validation of a base line model derived from the scale validation stage in order to determine stability of estimates across two independent samples of students. Multiple-sample analyses allow the researcher to constrain model parameters to be identical across two or more samples and to test how well these constraints fit the data [39]. The unconstrained base line model was compared with 3 other CFA models that were constrained at increasingly stringent levels (see Table 3). This comparison was intended to evaluate whether the factors, the correlation between the two factors, and the items in each factor were consistent across Group 1 and Group 2 [39]. Indicators to evaluate the equivalence of a model across groups which rely on differences of *chi square* in each model are influenced by the sample size [39] and thus problematic. Therefore, we used the criterion based on a difference in CFI (ΔCFI) of less than 0.01 [40].

## Results

### Scale validity

**Fig. 2:**
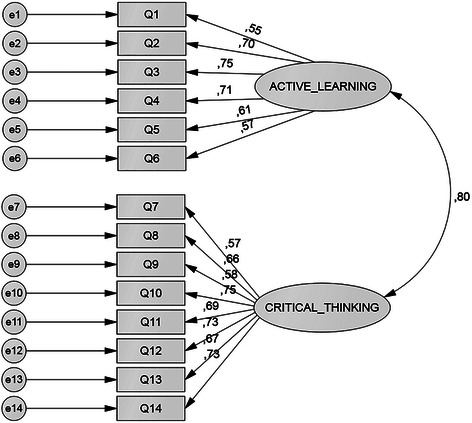
The two-factor model of the self-assessment tool in the PBL tutorial

The CFA of the two-factor model with 29 items indicated that this model had a poor fit based on the fit indices recommended by AMOS^TM^. Some items had high correlations with other items in the differing factors. To obtain a model fit, all of these items were removed. As a result, a two-factor model consisting of 14 items was a close fit to the data in Group 2 (See Fig. 2), due to the fact that all of the fit indices criteria, which were used in data analysis, were fulfilled. The results were CMIN/df = 1.99, p = 0.000, CFI = 0.94, TLI = 0.93, GFI = 0.92, AGFI = 0.88, R = 0.06. The loading factor for each item was > 0.50 with a smallest value of 0.55 (item no 1) and the highest of 0.75 (item no 3 and 10) (see Fig. 2). However, the other finding from this analysis showed that the correlation between the two factors was fairly high (r = 0.80), indicating that this tool was derived from a potential single underlying factor. To check this, the one factor solution model was compared with the two factors solution model [39]. The resulting parameters such as the value of CFI, TLI, GFI, AGFI, CMIN/df and RMSEA indicated that the two-factor solution model was better and more appropriate to the data than the one factor solution model. The values of CFI, TLI, GFI, and AGFI of the two factors model were higher than one factor model, and the values of CMIN/df and RMSEA of the two factors model were lower than the other one. The comparison of fit indices between these two models is given in Table 1.

**Table 1:** The fit indices for the one and two-factor models from Group 2 data

Number of factors	Number	X^2^	df	P	CMIN/df	RMSEA	TLI	CFI	RMR	GFI	AGFI
1	238	224.65	77	0.000	2.911	0.090	0.864	0.885	0.095	0.864	0.814
2	238	151.61	76	0.000	1.995	0.065	0.929	0.941	0.069	0.915	0.882

Cronbach’s alpha showed that the reliability coefficient was more than 0.8 for each factor (see Table 2), meaning that each subscale scale had good internal consistency. Table 2 also provides the mean and standard deviation for each factor.

**Table 2:** Number of items, number of students, mean, standard deviation and the Cronbach alpha for each factor and the scale from Group 2 data

Factors	n items	n students	Mean	SD	Alpha
Critical Thinking	8	238	4.8	1.3	0.84
Active learning	6	238	5.4	1.1	0.81
Total	14	238	5.1	1.3	0.89

### Scale stability

The result of the multi group analysis across Group 1 and Group 2 indicated that the base line model, a two-factor model with 14 items had acceptable fit indices. The CFI and RMSEA were 0.923 and 0.051 respectively. Subsequently, this baseline model was compared with the three other models which each had increasingly stringent invariance constraints (see Table 3). The multi group analysis showed that the difference in CFI (ΔCFI) between the base line model and each of the 3 other models was less than 0.01. The differences in CFI (ΔCFI) were 0.003, 0.003 and 0.008 for model A, model B, and model C respectively. This result indicated that Group 1 and Group 2 had the same structural model including similarity in the factorial structure, the theoretical construct, and structural regression paths. The base-line model consisting of 2 factors with 14 items was quite stable across two independent samples since there were no significant differences with the 3 other models.

**Table 3:** Multi-group analysis of Group 1 and Group 2 for the measurement invariance

Model	X^2^	df	ΔX^2^	Δdf	TLI	CFI	Δ CFI	GFI	AGFI
Baseline model (No constraint model)	346.183	152	-	-	0.908	0.923	-	0.906	0.870
Factor loading invariant (Model A)	367.276	164	21.093	12	0.911	0.920	0.003	0.901	0.874
Factor loadings and factor correlations invariant (Model B)	370.805	167	24.622	15	0.912	0.920	0.003	0.9	0.875
Factor loadings. factor correlations, and factor invariance invariant (Model C)	396.968	181	50.785	29	0.914	0.915	0.008	0.895	0.878

## Discussion

This paper has provided evidence of the validity of the SSACT by demonstrating its content validity, and its internal structure. All of the processes from defining the domain, constructing the definition, developing the items, and conducting expert reviews provided evidence of content validity [20–22].

After condensing, the final scale consisted of 2 factors with 14 items (see Table 4). The CFA confirmed that this scale consisted of a two dimensional construct. Each item in this scale was derived from its latent construct or first order factor. The factors underlying the structure of SSACT were active learning and critical thinking. The factor structure appeared stable across independent student populations [39].

**Table 4:** The final version of Self-Assessment Scale on Active Learning and Critical Thinking (SSACT)

No	Items	Factors/Subscales
1	I set my own learning objectives for each scenario, in addition to the group objectives.	Active Learning
2	I applied various learning strategies during independent study.	
3	I was able to summarize the key points of the outcome of the group discussion.	
4	I managed my independent study effectively.	
5	My behaviour encouraged other members to actively participate in the tutorial process.	
6	I reflected on my learning in each scenario based on the objectives that I set myself.	
7	I was able to formulate questions based on the scenario.	Critical Thinking
8	I communicated my ideas clearly.	
9	I performed the role given to me by other group members.	
10	In the second meeting, I applied knowledge from my independent study to provide a solution to the problem being discussed.	
11	I analysed information in the scenario using relevant theory and concepts.	
12	I made links during the tutorial process between my newly acquired knowledge and my previous knowledge.	
13	I explained knowledge from the resources in my own words.	
14	I could generate a hypothesis to explain the problem under discussion.	

The active learning scale consisted of items related to collaborative learning (item no 5) and self directed-learning skills (item no 1–4, 6) (Table 4). These results are in line with Yew et al. [41], who also found that active learning in PBL was a cumulative process and was empowered by collaborative learning and self-directed learning processes. On the other hand, the inclusion of critical thinking as one of the domains in this scale also provided evidence that PBL tutorials stimulate students’ critical thinking. In PBL, students actively construct their knowledge through elaboration process [19, 32, 42]. All of the items in the critical thinking domain (Table 4) including item no 8, 9 and 13, reflect on cognitive skills applied during PBL tutorial such as questioning, analysing, and generating hypothesis. Item no 8 “I communicated my ideas clearly” includes skills in organizing ideas [43]. Item 9 “I performed the role given to me by other group members” means that each student has same responsibility to participate actively in PBL tutorial by explaining and questioning, which are forms of critical thinking [44, 45]. Item 13, “I explained knowledge from the resources in my own words” is related to the skill of paraphrasing where students need to understand the information first before they explain it to others [43].

The strong significant correlation between the two subscales (r = 0.80) was evidence that the SSACT has two distinct but theoretically strongly related constructs [28]. Critical thinking is a self-directed process to assist connecting new knowledge to prior knowledge. Students need to justify their new understanding through sharing with others. On the other hand, self-directed learning referred to students’ internal cognitive processes in managing their learning in order to achieve their learning goals. To be self-directed learners, students need to think critically regarding their own learning condition [46].

Our data also indicated that the SSACT had good reliability for each subscale as well as total scale (coefficient Alpha >0.8). This means that the interrelatedness among the designed items in each subscale supported its construct and all items in the scale also supported the self- assessment construct [28].

### Strengths and weaknesses of the research

The strength of this study is that we applied a number of complementary strategies in developing the tool. Previous studies of PBL self-assessment tool development constructed the domains from literature review or previous existing tool [12, 14]. In this study, the domains were derived from student and tutor perspectives of a PBL tutorial captured via a qualitative study. The final items and domains of the SSACT reflected students’ activities during problem analysis, self- directed learning and the reporting phase in the PBL tutorial. The SSACT also had good construct, internal consistency and stability when applied across samples. All of these results suggest that SSACT is appropriate for the PBL context.

On other hand, this study also had some limitations. So far, the impact of this tool on students learning, a part of the consequential validity, has not been explored. The relationship of the SSACT to other variables of interest such as academic achievement, quality of learning environment and students’ motivation also has not been identified [20–22]. Furthermore, the original scale was developed in Bahasa Indonesia and validated in Indonesia. Even though the English version was developed together with the original version, the Indonesian research context and student characteristics might influence the result of the validation for an English language PBL context.

### Implications and future research

Students could use this tool to guide their learning in several ways. Firstly, use of this tool may enable students to better understand the aspects that should be considered in each phase of the PBL tutorial. These include the problem analysis phase, the self-directed learning phase, and the reporting phase. Secondly, the active learning subscale could inform the students on how to become better self-directed learners. This tool asks the students about their learning activities in order to identify their learning needs, apply appropriate learning strategies, monitor their progress, and evaluate their performance. Thirdly, the critical thinking subscale could alert the students to the need to conduct higher order cognitive activities and may reduce superficial thinking. Engagement in both active learning and critical thinking activities may help to prevent PBL erosion since these activities are the core processes of PBL tutorial [2]. When applied across multiple PBL cases, the SSACT may also stimulate students to become better self-regulated learners since self- assessment could facilitate students to monitor their learning and identify their own strengths and weaknesses and form a basis on which to improve their learning [47].

In order to optimise the benefits of this self-assessment scale, students need scaffolding from both the tutor and faculty in applying this scale. Students should be informed the advantages of self-assessment in assisting their learning. By understanding the objectives of self assessment, students would be more likely to assess themselves honestly and be more motivated to improve areas of weaknesses in their learning [48, 49]. Constructive feedback from the tutor will also promote student learning and achievement through self-assessment [48]. This scale is intended to be implemented formatively. If self-ratings were used summatively, students could not assess themselves in a reliable manner and would tend to report good behaviours in order to obtain good mark [49].

Future research could be conducted to investigate further evidence of validity by comparing this tool with other measurements of critical thinking and active learning and by evaluating the educational impact. Research in other settings, such as other medical schools and countries with different cultures and different PBL process will provide more information about the broader utility of the self-assessments tool.

## Conclusion

The development and the validation process of the SSACT provides evidence regarding its internal structure, which was investigated through its factorial validity, scale stability and internal consistency. The two factors underlying this scale, active learning and critical thinking, were important skills that should be central to a PBL tutorial. Implementation of this self-assessment scale in a PBL tutorial may contribute to guiding students to achieve the essential outcomes of the PBL method and may stimulate them to become self-regulated learners.
